# Sero-surveillance for SARS-CoV-2 infection among healthcare providers in four hospitals in Thailand one year after the first community outbreak

**DOI:** 10.1371/journal.pone.0254563

**Published:** 2021-07-14

**Authors:** Wanitchaya Kittikraisak, Phunlerd Piyaraj, Apichat Vachiraphan, Thanapat Wongrapee, Somsak Punjasamanvong, Taweewun Hunsawong, Chalinthorn Sinthuwattanawibool, Chaniya Leepiyasakulchai, Pornsak Yoocharoen, Eduardo Azziz-Baumgartner, Joshua A. Mott, Suthat Chottanapund

**Affiliations:** 1 Influenza Program, Thailand Ministry of Public Health – U.S. Centers for Disease Control and Prevention, Nonthaburi, Thailand; 2 Phramongkutklao College of Medicine, Bangkok, Thailand; 3 Bamrasnaradura Infectious Diseases Institute, Nonthaburi, Thailand; 4 Phaholpolpayuhasena Hospital, Kanchanaburi, Thailand; 5 Rayong Hospital, Rayong, Thailand; 6 Virology Department, Armed Forces Research Institute of Medical Sciences, Bangkok, Thailand; 7 Faculty of Medical Technology, Mahidol University, Nakhon Pathom, Thailand; 8 Department of Disease Control, Ministry of Public Health, Nonthaburi, Thailand; 9 Influenza Division, U.S. Centers for Disease Control and Prevention, Atlanta, Georgia, United States of America; All India Institute of Medical Sciences Jodhpur, INDIA

## Abstract

**Background:**

Thailand was the first country outside China to report SARS-CoV-2 infected cases. Since the detection of the first imported case on January 12^th^, 2020 to the time this report was written, Thailand experienced two waves of community outbreaks (March-April 2020 and December 2020-March 2021). We examined prevalence of SARS-CoV-2 seropositivity among healthcare providers (HCPs) in four hospitals approximately one year after SARS-CoV-2 first detected in Thailand. By March 2021, these hospitals have treated a total of 709 coronavirus disease 2019 (COVID-19) patients.

**Methods:**

Blood specimens, collected from COVID-19 unvaccinated HCPs during January-March 2021, were tested for the presence of SARS-CoV-2 immunoglobulin G (IgG) antibodies to nucleocapsid (IgG-nucleocapsid) and spike (IgG-spike) proteins using Euroimmune^®^ enzyme-linked immunosorbent assays.

**Results:**

Of 600 HCPs enrolled, 1 (0.2%) tested positive for the SARS-CoV-2 IgG-spike antibodies, but not the IgG-nucleocapsid.

**Conclusion:**

The presence of SARS-CoV-2 IgG antibodies was rare in this sample of HCPs, suggesting that this population remains susceptible to SARS-CoV-2 infection.

## Introduction

Since detected in December 2019, a novel coronavirus (SARS-CoV-2) has spread rapidly, causing substantial morbidity and mortality worldwide. Occupational exposures among healthcare providers (HCPs) have been documented in many countries [1]. The risk of SARS-CoV-2 infection among HCPs is high with estimated attack rate among HCPs as high as 30% [2].

Thailand was the first country outside China to report SARS-CoV-2 infected cases. Since the detection of the first imported case on January 12^th^, 2020 to the time this report was written, Thailand experienced two waves of community outbreaks (March-April 2020 and December 2020-March 2021). By March 2021, of 1,663,330 specimens tested, 27,876 (2%) real-time reverse transcription polymerase chain reaction (rRT-PCR)-confirmed cases have been found in 76 of 77 provinces [3]. Per Thailand’s regulations, identified cases are required to stay in healthcare facilities until testing negative by the rRT-PCR. The risk of SARS-CoV-2 infection within Thai healthcare settings is unclear. While HCPs represent 0.3% of the population, they represent 0.07% of SARS-CoV-2 infected cases [3]. Approximately 50% of the infection was thought to occur because of occupational exposures; the highest proportion of HCP infection occurred among nurses [3].

Most persons infected with SARS-CoV-2 display an antibody response between day 10 and day 21 after infection. The presence of antibodies is detected in <40% among patients within one week from onset, and rapidly increases to 100% for total antibodies, 94% for immunoglobulin (Ig) M, and 80% for IgG at day-15 after onset [4]. Antibody response in mild cases can take longer (≥4 weeks); and in a small number of cases antibodies are not detected at all (at least during the studies’ time scale) [5]. Currently, the longevity of the antibody response to SARS-CoV-2 is being studied, but it is known that antibodies to other coronaviruses wane 12–52 weeks from the onset of symptoms, potentially placing persons at risk for re-infection [6]. However, SARS-CoV-2 IgM and IgG antibody levels have been shown to remain over the course of seven weeks [7] or in 80% of the cases at day 49 post onset [8]. In comparison, 90% of patients infected with the related SARS virus, namely SARS-CoV-1, may maintain IgG antibodies for two years [9].

In January 2021, we began a prospective cohort of Thai HCPs to estimate the prevalence of antibody seropositivity, seroconversion proportion, antibody functional immunity, cellular immune response, and the cumulative incidence to SARS-CoV-2 infection. These data may provide an understanding about the disease burden in HCPs and help track acceleration/deceleration of the epidemic in response to specific mitigation policies that healthcare settings have implemented. This report details the prevalence of SARS-CoV-2 seropositivity, as measured by enzyme-linked immunosorbent assay (ELISA), among COVID-19 unvaccinated participants at baseline blood collection.

## Materials and methods

### Study design and setting

A cohort of HCPs was established at: Phramongkutklao Hospital (PMK, 1,200 beds, total 1,428 HCPs); Bamrasnaradura Infectious Diseases Institute (BIDI, 300 beds specializing in infectious diseases, total 819 HCPs); Phaholpolpayuhasena Hospital (PH, 540 beds, total 1,400 HCPs); and Rayong Hospital (RY, 567 beds, total 1,600 HCPs) for a 1-year follow-up. PMK and BIDI serve populations in the Bangkok Metropolitan area, while PH and RY serve those in Thailand’s Western and Eastern regions, respectively. All have treated COVID-19 cases (97 at PMK, 259 at BIDI, 13 at PH, and 340 at RY). The HCPs, defined as individuals providing direct healthcare services (e.g., vital sign measurement, bathing or examining patients, taking specimens from patients, etc.) in a healthcare setting, aged ≥18 years, worked ≥30 hours/week, and having had potential occupational risk to SARS-CoV-2 infection (determined by reported work location, ≥1 patients cared for per day, and the assigned duties) were enrolled by convenience sampling. Those who were employed <1 year or acutely ill with COVID-19 at enrollment were excluded.

At enrollment, data on demographic, health and work history, potential occupational and non-occupational risks, and personal protective and risk behaviors were collected using standardized questionnaires. In addition, participants were inquired about number of suspected and confirmed SARS-CoV2 cases encountered and the locations. They then self-assessed risk of SARS-CoV-2 infection in various situations on a scale of 0 (lowest) to 10 (highest). During weekly surveillance, those reporting ≥1 of: myalgia, cough, runny nose/nasal congestion, sore throat, or difficulty breathing self-collected nasal swabs for SARS-CoV-2 rRT-PCR testing using the U.S. CDC’s protocol with high sensitivity and specificity for detecting 5 RNA copies/reaction with no observed false-positive reactivity [10, 11]. At enrollment, 30% of participants were randomly selected as an asymptomatic subgroup for weekly self-nasal swab collection for SARS-CoV-2 rRT-PCR testing. During the enrollment process, study staff taught all participants how to self-collect nasal swabs by an in-person demonstration. Participants were then given kits for self-swab collection and instruction for storage. Blood specimens were collected from all participants at enrollment for antibody serology testing.

### Baseline blood collection and processing

Heparinized blood specimens (10 mL) were collected and transported to Mahidol University’s laboratory within five hours of collection. The separated plasma was kept in -20 °C until transported on dry ice to the Armed Forces Research Institute of Medical Sciences’ laboratory. All baseline blood specimens were collected prior to the HCPs having received COVID-19 vaccines.

### Laboratory testing

The plasma was thawed and heat-inactivated (56 °C for 30 minutes). Studies have demonstrated that heat inactivation does not decrease the detection efficacy of SARS-CoV-2 IgG antibodies [12, 13]. In batch, plasma was screened for the presence of SARS-CoV-2 IgG antibodies to nucleocapsid (IgG-nucleocapsid) and spike (IgG-spike) proteins using the Thai Food and Drug Administration approved Euroimmune kits^®^ (Lübeck, Germany) following the manufacturer’s instructions. Per the manufacturer, the sensitivity and specificity of the assay for IgG-nucleocapsid are 80% and 100%, respectively, for specimens collected <10 days post illness onset, and 95% and 100%, respectively, for specimen collected ≥10 days post illness onset. The sensitivity and specificity of the assay for IgG-spike are 44% and 100%, respectively, for specimens collected <10 days post illness onset, and 94% and 100%, respectively, for specimens collected ≥10 days post illness onset. Positive and negative controls were included in all assay batches. To test, diluted plasma was incubated in reaction wells each coated with recombinant SARS-CoV-2 structural protein. For a positive specimen, specific IgG antibodies that bound to the antigens were detected using an enzyme-conjugated colorimetric technique. Semi-quantitative results were calculated as a ratio of the extinction of the control or tested specimen over the extinction of the calibrator. Ratio values of <0.8 were considered as negative, ≥0.8 to <1.1 as borderline, and ≥1.1 as positive. For the quantitative kits that included six calibrators for quantification of the antibody concentration, results were reported in standardized units (i.e., binding antibody units/mL).

### Data analysis

Frequency and median were calculated to describe participants’ characteristics. Seropositivity was calculated by dividing number of ELISA positive cases by the number of all enrolled HCPs. Analyses were conducted using Stata, version 16 (Stata Corp., USA).

### Ethical approval and informed consent

This study was approved by the Institutional Review Boards (IRBs) of: the PMK (Thailand); BIDI (Thailand); PH (Thailand); RY (Thailand); Department of Disease Control of the Thai Ministry of Public Health (MOPH; Thailand); and Walter Reed Army Institute of Research (USA). The IRB of the U.S. Centers for Disease Control and Prevention (USA) and Mahidol University (Thailand) relied on the determinations of PMK’s and MOPH’s IRBs, respectively. All participants had provided written informed consent.

## Results

### Study participants’ characteristics

During January 18^th^, 2021-March 5^th^, 2021, we enrolled 600 Thai HCPs (310 from PMK, 120 from BIDI, 100 from PH, and 70 from RY). The median age was 39 years (interquartile range [IQR], 29–47); the majority (548; 91%) were female (Table 1). Among these, 390 (65%) were nurses, 119 (20%) were nurse aids, 19 (3%) were physicians, 12 (2%) were laboratorians, and 60 (10%) had other professions. Two hundred and fifty-six (43%) worked in an outpatient department, 258 (43%) in an inpatient department, and 86 (14%) worked in both departments. The median employment duration at the study facility was 12 years (IQR, 5–24). Two hundred and three (34%) participants had pre-existing medical conditions. The most common conditions were metabolic disease including diabetes (85; 14%), hypertension (54; 9%), and bone disease e.g., osteoporosis, arthritis (11; 2%; Table 2).

**Table 1:** Characteristics of study participants at enrollment (January-March 2021), Bangkok, Thailand.

Characteristics	Number of participants (%)
Age (years)	39 (29–47)*
Sex	
Male	52 (9)
Female	548 (91)
Profession	
Nurse	390 (65)
Nurse aid	119 (20)
Physician	19 (3)
Laboratorian	12 (2)
Others**	60 (10)
Work location	
Outpatient	256 (43)
Inpatient	258 (43)
Outpatient and inpatient	86 (14)
Employment duration at study facility (years)	12 (5–24)*
Number of pre-existing medical condition	
Not sure	3 (<1)
0	394 (66)
1	154 (26)
2–4	49 (8)

*Median (interquartile)

**Assistant to dentist, triage staff, technician for coronary artery angiography, technician at sport medicine center, and paramedic.

**Table 2:** Pre-existing medical conditions among the study participants who reported at least one condition at enrollment.

Condition	Number (%)
Metabolic disease (including diabetes)	85 (14)
Hypertension	54 (9)
Bone disease e.g., osteoporosis, arthritis	11 (2)
Heart and circulatory disease (excluding hypertension)	9 (1)
Asthma	8 (1)
Liver disease	7 (1)
Hemoglobinopathy including thalassemia	5 (1)
Autoimmune disease	5 (1)
Neurologic/neuromuscular disorder (including muscular dystrophy, cerebral palsy)	4 (1)
Cancer	3 (<1)
Obesity	3 (<1)
HIV infection	2 (<1)
Others*	12 (2)

*Vertigo, depression, vitiligo, glaucoma, gastroesophageal reflux disease, and ovarian endometrioma

### Exposure risk to SARS-CoV-2 suspected/confirmed cases

Seven days prior to enrollment, 81 (13%) participants self-reported close contact, defined as conversing in close proximity but with no skin-to-skin contact, with SARS-CoV-2 suspected cases, 8 (1%) with confirmed cases, and 21 (3%) with both suspected and confirmed cases in occupational and non-occupational settings. Of those with reported exposures, the median number of suspected and confirmed cases encountered were 5 (IQR, 2–15) and 3 (IQR, 1–10), respectively. Most encounters occurred in the workplace, as reported by 104 (95%) participants, while the rest occurred in the community (2; <1%), in household (1; <1%), or in all three locations (3; 3%). Forty-three (39%) of 110 participants also reported having a physical contact with suspected/confirmed cases. From a 10-point scale, perceived risk levels of SARS-CoV-2 infection were highest from occupational exposure (median, 5; IQR, 3–7) and travelling (median, 5; IQR, 2–7), followed by from the community (median, 4; IQR, 2–5), and in the household (median, 1; IQR, 0–3).

### Sero-prevalence of SARS-CoV-2 infection

SARS-CoV-2 IgG antibodies were found in 1 (0.2%) of 600 participants. The ELISA ratio values for this person were 0.02 for IgG-nucleocapsid (i.e., considered negative) and 1.6 for IgG-spike (i.e., considered positive) using the semi-quantitative kits. The same specimen tested positive for IgG-spike with antibody level of 84 binding antibody unit/mL using the quantitative kit. Repeated testing using the same specimen yielded the same interpretation. Subsequent testing of a blood specimen collected two weeks later, however, was negative for IgG-nucleocapsid and IgG-spike antibodies. Serial blood specimen collected from this questionable individual at month 3 (92 days from the first blood collection and 21 days after receipt of one dose of the Sinovac^®^ vaccine) was positive for IgG-spike (154 binding antibody units/mL) but negative for IgG-nucleocapsid antibodies. The amount of IgG-spike antibodies in month 3 blood specimen from this individual was higher than that of others receiving one and two doses of the same COVID-19 vaccine who had blood specimens collected at 21 days post vaccination (median, 17 binding antibody units/mL; IQR, 8–28 binding antibody units/mL for one dose; median, 133 binding antibody units/mL; IQR, 107–203 binding antibody units/mL for two doses, respectively).

## Discussion

Despite two waves of community outbreaks (during March-April 2020 and December 2020-March 2021) with all identified cases being cared for within the healthcare setting (including our study facilities), the presence of SARS-CoV-2 IgG antibodies was rare in a sample of COVID-19 unvaccinated HCPs in four Thai hospitals. This finding suggests that unvaccinated HCPs remain susceptible to SARS-CoV-2 infection during the on-going pandemic.

In this study, only one IgG-spike ELISA positive case was identified a year after the initial wave. The low prevalence in this study is consistent with three sero-surveys conducted in the country which reported the IgG prevalences of <1%. In July-September 2020 after the initial wave, only one (0.01%) of 6,651 army personnel tested positive for SARS CoV-2 antibodies [14]. Similarly, two surveys conducted during April-May 2020 and April-June 2020 among HCPs in hospitals that did not treat COVID-19 cases reported IgG prevalences of 0% and 0.2%, respectively [15, 16]. Based on available data, antibodies to SARS-CoV-2 may develop between 6–15 days after infection and are generally measurable by week 2–3 [4, 17]. Studies have also reported that milder infection may not produce the strong and long-term immunity provided by specific antibodies against SARS-CoV-2.(5).

Since the start of the pandemic in 2020, Thailand has implemented several infection control measures, all of which have been widely adopted in both healthcare and community settings [3]. Findings from this study and the other sero-surveys conducted in Thailand suggest that to date there has been relatively effective control of SARS-CoV-2 transmission in both healthcare setting and in the community.

A strength of this study is that all of our study facilities are designated as primary locations for caring of the country’s COVID-19 cases. Being a referral hospital specializing in infectious diseases, the BIDI received most of the initial COVD-19 cases during the first epidemic wave in the country [18], suggesting that the HCPs at BIDI had high likelihood for contact with COVID-19 cases. As the pandemic evolved and the country expanded its resources, other facilities began to receive COVID-19 cases as well. Additionally, Rayong province was a focal point for new infection at the beginning of the country’s second epidemic wave, to which the RY had served as the primary facility caring for identified cases. Another strength is that all blood specimens were collected from COVID-19 vaccine naïve HCPs at the time, giving us the opportunity to determine the extent of natural SARS-CoV-2 infection in the HCP population. Our findings represent a sample of HCPs in four facilities, however, and not the entire Thai healthcare system. Although we enrolled a variety of medical professions, enrollment was based on convenience sampling and the numbers of laboratorians and physicians participating in the study are relatively low. Lastly, it is not known if the low prevalence surveyed at one point in time in this study reflects low infection among the HCPs or the waning of immunity [19, 20].

Our data indicate a near zero rate of SARS-CoV-2-specific IgG seropositivity in HCPs in four Thai hospitals that have been assigned as treatment facilities for COVID-19 cases, suggesting that this population remains susceptible to SARS-CoV-2 infection. This finding may represent the effective prevention of SARS-CoV-2 transmission within these healthcare facilities. As COVID-19 vaccines are now being distributed to various risk groups including these frontline individuals, continued follow-up of the cohort participants may aid in monitoring the pandemic, identifying risk factors for HCP infection, and assessing the quality of immune responses elicited by vaccination in this population.
